# Percutaneous Balloon Compression for the Treatment of Trigeminal Neuralgia: A Review of 10 Years of Clinical Experience

**DOI:** 10.7759/cureus.43645

**Published:** 2023-08-17

**Authors:** Bayron Valenzuela Cecchi, Francisca Figueroa, Luis Contreras, Patricio Bustos, Felipe Maldonado

**Affiliations:** 1 Department of Neurology and Neurosurgery, Hospital Clínico de la Universidad de Chile, Santiago, CHL; 2 Department of Neurosurgery, Universidad de la Frontera, Temuco, CHL; 3 Department of Anesthesiology and Perioperative Medicine, Hospital Clínico de la Universidad de Chile, Santiago, CHL

**Keywords:** balloon rhizotomy, meckel’s cave, pain, trigeminal neuralgia, percutaneous balloon compression

## Abstract

Background: Trigeminal neuralgia (TN) is defined as a spontaneous painful sensation in the trigeminal nerve territory. The pain intensity of TN is classified into different grades of suffering that affect a patient’s quality of life. Percutaneous balloon compression of the ganglion is a neurosurgical option that is easy, reproducible, and can reduce the morbidity of TN.

Methods: We retrospectively analyzed all patients treated with trigeminal nerve percutaneous balloon compression at the Clinical Hospital of the University of Chile between January 2012 and May 2022. Data collected from electronic records included demographic information, medical and surgical history, type of anesthesia and drugs used during surgery, balloon inflation time, surgery time, operative room time, intraoperative events, postoperative complications, duration of hospitalization, and duration of follow-up.

Results: We identified 63 patients who met our inclusion criteria. The median patient age was 62 years (interquartile range [IQR] 57-69 years). Sixty-five percent of the patients were female. The simultaneous involvement of the second and third branches of the trigeminal nerve was the most frequent symptom. Before surgery, the patients experienced an average of 6.6 years of pain (IQR 2-10 years). Right neuralgia was the most frequent laterality type (69%). Forty percent of the patients had a previous surgical procedure for neuralgia, with treatment failure being the most frequent surgical indication (63%). According to the procedure, the mean balloon insufflation volume was 0.89±0.12 mL with a median compression time of 2.5 min (IQR 2.1-4.0 min). No hemorrhagic complications were observed. Furthermore, during follow-up, there were no surgical complications among any of the patients; however, 6.4% of patients required a second intervention. The pain-free period was two years in 60% of patients and five years in 23% of patients.

Conclusions: TN is a painful condition. Although there are multiple surgical approaches, we believe that percutaneous balloon compression is an excellent alternative treatment option that offers high effectiveness, low morbidity, and low hospital stay.

## Introduction

Trigeminal neuralgia (TN) is defined as a spontaneous painful sensation in the trigeminal nerve territory. Pain intensity involves different grades of suffering that affect the quality of life. The main criteria for diagnosis are brief, intense, and sudden paroxysms of pain triggered by innocuous stimuli such as talking, chewing, or light touch of the overlying skin, and is restricted to one or more branches of the trigeminal nerves [1]. Although TN is a rare condition, affecting 4-13 people per 100,000 annually, it is the most common facial neuralgia. It is more common in women (2:1), with an incidence that increases with age and is more frequent in the fifth or sixth decade of life [2-4].

Treatment options include medications and surgery. The initial management of choice for TN is medical pharmacotherapy. Patients who do not tolerate or fail medical management may benefit from surgery [3,4]. The options are microvascular decompression (MVD) of the trigeminal nerve root, rhizotomy percutaneous techniques (radiofrequency thermocoagulation, mechanical balloon compression, and chemical neurectomy), peripheral technical, radiosurgery, and deep brain stimulation [2,5].

In general, all techniques have an initial response of more than 80% at five years, and MVD has a superior response compared to the other techniques. Significant risks associated with MVD occur at rates of less than 2% and include stroke, meningitis, and death [2,5].

Percutaneous balloon compression (PBC) of the Gasserian ganglion (GG) is an alternative neurosurgical treatment option. The technique involves needle insertion through the foramen ovale under the GG. With the needle tip placed in Meckel's cave, a Fogarty catheter is inserted, and balloon insufflation with contrast material is performed under direct radioscopic vision. The balloon is loaded with up to 1 mL of the solution, compressing the ganglion for 1.3-3 minutes (Mullan technique). Compression of trigeminal fibers allows for immediate pain relief in 80-90%. Advantages of this technique include high effectiveness, a low hospital stay, and minimal morbidity and mortality [1,4].

In 1991, we started treating TN using PBC of the GG. Here, we present the results of a 10-year retrospective review (2012-2022) at the Clinical Hospital of the University of Chile.

This article was presented as a poster at our Chilean neurosurgical meeting in September 2022.

## Materials and methods

Study design and setting

A retrospective study was conducted using digitized records of patients who underwent PBC of the trigeminal nerve at the Clinical Hospital of the University of Chile between January 2012 and May 2022. The study received approval from the ethics committee (Ref: OAIC:33/2022). All personal information was handled confidentially.

A search was conducted within the electronic clinical records of all surgeries identified using the terms "Percutaneous balloon compression" and "Neurolysis with microsurgical technique." Patients who underwent alternative procedures, including peripheral neurectomies, percutaneous glycerol rhizotomy, percutaneous radiofrequency rhizotomy, radiosurgery, and MVD, were excluded from the analysis.

Data collected from electronic records included demographic information, medical and surgical history, type of anesthesia and drugs used during surgery, balloon inflation time, surgery time, operative room time, intraoperative events, postoperative complications, duration of hospitalization, and follow-up time of patients (Table 1).

**Table 1:** Baseline characteristics of study participants IQR, interquartile range.

Age	
Median (IQR)	62 (57-69)
Range	58
Sex - n/total n (%)	
Male	22/63 (35)
Female	41/63 (65)
Nerve territory (n)	
V1-V2-V3	1
V2-V3	41
V1-V2	6
V1	0
V2	9
V3	5
Clinical neuralgia	
Left neuralgia	19 (31)
Right neuralgia	23 (69)
Pain duration (median in years)	
Years, IQR (range)	6.65, 2-10 (24.7)
Prior surgery	
Yes (%)/No (%)	18 (40)/45 (60)
Surgical indication n (%)	
Recurrence	16 (25)
Treatment failure	40 (63)
No Information	7 (11)
Balloon insufflation	
Mean volume (mL)	0.89
IQR (range)	0.8-1.0 (0.4)
Compression time	
Median (minutes)	2.5
IQR (range)	2.1-4.0 (9.8)
Complications (%)	
No	44 (70)
Anesthesia; paresthesia	1 (1.6); 2 (3.2)
Hypoesthesia	16 (25)
Comorbidities	
Hypertension	14/63
Diabetes mellitus, type 2	3/63
Insulin resistance	2/63
Dyslipidemia	1/63
Hypothyroidism	6/63
Hyperthyroidism	1/63
Chronic kidney disease	1/63
Polyarthralgia	1/63
Cardiac arrhythmias	2/63
Aortic valve replacement	1/63
Chronic airflow limitation	1/63
Colorectal cancer	1/63
Parathyroid cancer	1/63
Pituitary macroadenoma	1/63
Benign prostatic hyperplasia	1/63
Unipolar depression	2/63
Fibromyalgia	1/63
Hemifacial spasm	1/63
Hearing loss	1/63
Drugs	
Carbamazepine; oxcarbazepine	42; 1
Pregabalin	9
Gabapentin	2
Duloxetine	2
Tramadol	7
Acetaminophen	4
Baclofen	2
Botox	1

Quantitative variables and statistical methods

Categorical variables are summarized as relative frequencies. Continuous variables were expressed as means (and standard deviations) or medians (and interquartile ranges [IQR]). 

## Results

We found 63 patients treated with PBC within the 10-year lookback period (Table 1). The median patient age was 62 years (IQR 57-69 years). Sixty-five percent of patients were female. Out of the 63 surgeries performed, 21 of them were subsequent interventions for procedures previously conducted in another medical facility. After the brain MRI ruled out secondary causes of TN, all patients who underwent surgery were evaluated by a neurosurgical committee fulfilling the clinical criteria for performing the PBC procedure. The main surgical indication was medical treatment failure (63%), with carbamazepine (800 mg/day) being the most common drug administered.

The simultaneous compromise of the second and third branches of the trigeminal nerve (V2-V3) was the most frequent symptom, followed by the independent involvement of each of the territories. Patients were treated after 6.6 years of pain (IQR 2-10 years), with right neuralgia as the most common laterality (69%). Medical treatment failure was the most frequent surgical indication (63%), and in the 10-year study period, the recurrence rate was 25%.

All procedures were performed under general anesthesia. Propofol/remifentanil site-based target control infusion was the most frequent technical procedure for the administration and delivery of anesthesia.

**Figure 1:**
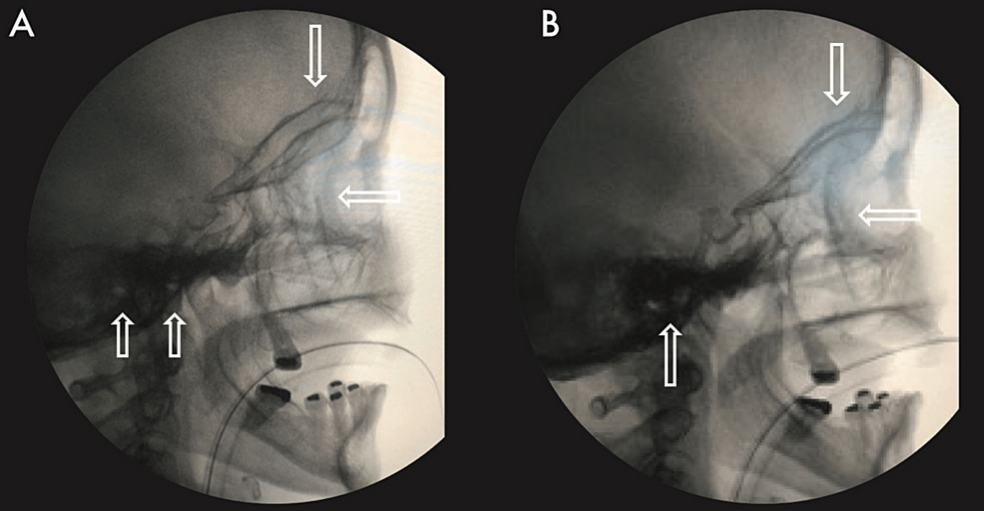
Radioscopic alignment control (sagittal view). A) No alignment. Upward arrow (EAC), downward arrow (ACF), leftward arrow (base of the pterygoid process). B) Alignment. The objective of this treatment is to achieve the best possible alignment. EAC, external auditory canal; ACF, anterior cranial fossa.

**Figure 2:**
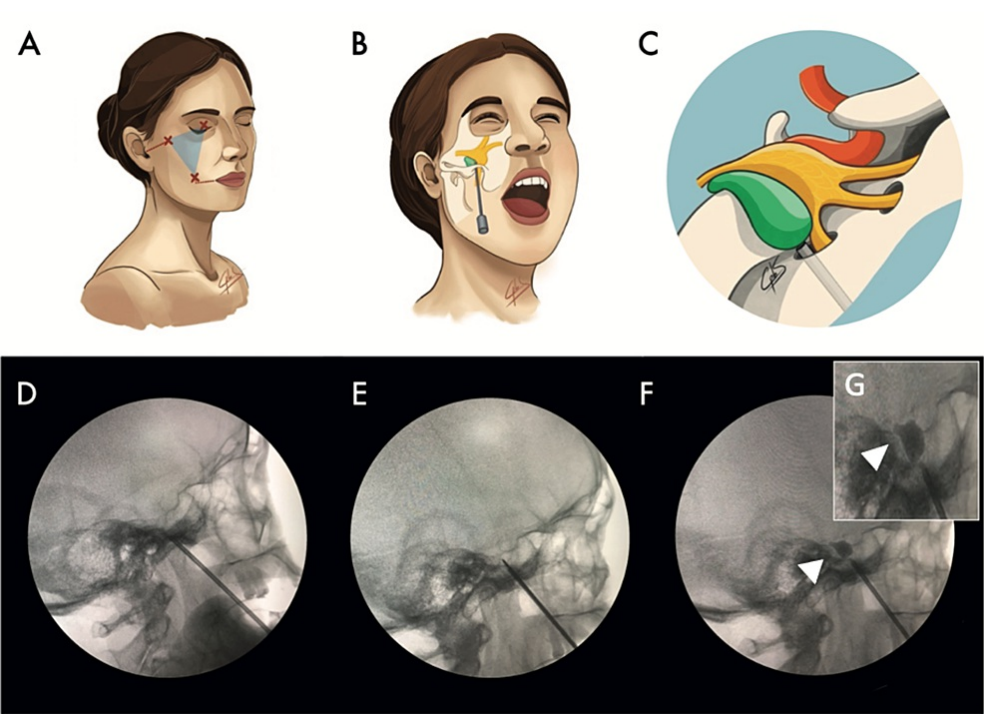
(A-C) Anatomic references (Mullan’s triangle). (D, E) The needle is directed to the foramen ovale. (F, G) Pear-shaped balloon in Meckel´s cave (white arrow head).

All surgeons performed the same procedure (Table 2 and Figures 1, 2). During the procedure, the mean balloon insufflation volume was 0.89±0.12 mL, with a median compression time of 2.5 min (IQR 2.1-4.0 min). During balloon insufflation, hypertension and bradycardia were the most frequent hemodynamic responses, thus requiring brief balloon deflation and atropine administration.

**Table 2:** Steps of procedure description

Steps
Step 1: General anesthesia: -Propofol/remifentanil target-controlled infusion. -Arterial line for direct arterial pressure monitoring (according to the patient’s comorbidities). -Atropine 0.5-1 mg bolus before Fogarty balloon insufflation.
Step 2: Patient positioning: -Supine with head in extension for radiographic viewing of the foramen ovale on fluoroscopy (lateral view) -Head fixation with tape
Step 3: Mullan triangle location: -2.5 cm lateral to the lip corner and 0.5 cm superior -Pupillary midpoint -3 cm anterior to the tragus/external auditory canal in relation to the orbitomeatal line -Objective: To reach the apex of the "pyramid,” access the foramen ovale, and reach Meckel's cavum
Step 4: Radioscopic alignment control (sagittal view): -Continuous fluoroscopy -External auditory canal alignment (EAC) -Pituitary fossa - clivus -Base of pterygoid process aligned -Anterior cranial fossa (ACF)
Step 5: Surgical stage: -Asepsis and antisepsis with Povidone Iodine Prep Solution -Large needle (trocar) -Nº4 Fogarty catheter -Tuberculin syringe -The needle is guided under fluoroscopic control until it engages the foramen ovale. The catheter is then gripped and advanced against the resistance to a depth of 1 cm. It should then be in Meckel's Cave. -Balloon insufflation (0.6-1 cc) with contrast material (visible). The balloon is distended until it begins to assume a pear shape, indicating that it is starting to protrude from the cave toward the posterior fossa. -Compression for 1.5-2.5 min -Radiography is performed for the record, and when the planned compression is achieved, the balloon is decompressed. The balloon and needle are then withdrawn. Firm digital pressure is applied to the skin of the cheek and maintained for 5 min to prevent hematoma formation.
Step 6: Post-surgical stage: -Assess full recovery of consciousness -Analgesic management -Neurologic evaluation

After surgery, only one patient presented with pain and 16 (25%) had hypoesthesia or paresthesia of the V2-V3 territory. The mean postoperative stay was one day. No hemorrhagic complications were observed in our case series.

Based on our records, during a median follow-up period of four months, no patient experienced any surgical complications; however, 10 patients presented with pain recurrence that required a second PBC procedure after 2.7 years.

Finally, a phone interview with the entire cohort was performed. Of the 42 patients who had their first surgery at our center, 29 responded to the telephone evaluation. Of these, 18 patients showed evolution to mild facial hypoesthesia. Additionally, three patients required a new surgery. Of these, one patient was pain-free for three months, one patient for one year, and one patient for two years before requiring a second surgery.

## Discussion

TN is a benign disease that does not modify a patient's life expectancy but has a meaningful impact on daily living activities. In this study, we reported our 10-year single-center experience with PBC of the GG for the treatment of TN.

Although TN is a rare condition, affecting 4-13 people per 100,000 annually, it is the most common facial neuralgia. It is more common in women (2:1), with an incidence that increases with age and is more frequent in the fifth or sixth decade of life [2-4]. Consistent with the literature, most cases in our case series were women (median age of 62 years) who had right neuralgia in the V2 and V3 territories (mean of 6.6 years of pain experienced).

Neurological examination and neuroimaging should exclude secondary causes, such as vascular malformations, multiple sclerosis, or expansive processes (secondary TN). Angio-magnetic resonance allows anatomical structure visualization of the trigeminal nerve and its relationships and is especially useful for ruling out neurovascular compression that may lead to posterior fossa surgical exploration, particularly in younger patients [3,6-8].

The pathophysiological origin of classic and secondary TN is nerve damage at its entry into the pons, and the trigeminal nerve (like all peripheral nerves) loses its Schwann cell myelin sheath, which is replaced by central myelin generated by oligodendrocytes. This transition zone is vulnerable to damage, particularly demyelination. Demyelination at these sites has been demonstrated by neurophysiological, neuroimaging, and histological studies [9]. When the myelin sheath becomes thin enough to allow the transmembrane passage of ions into the underlying axon, the axon is not equipped to rapidly pump out sodium. The resulting depolarization causes the axon to be hyperexcitable, leading to ectopic generation of high-frequency impulses after discharge (discharges that occur after the termination of the stimulus) and cross-fiber communication (called ephaptic transmission) [3,10-12].

Pharmacological and surgical management are used as TN treatment modalities (Table 3). Medical treatment as a first-line intervention was successful in 90% of patients. The first-line anticonvulsant drugs, carbamazepine (200-1200 mg/day) and oxcarbazepine (300-1800 mg/day), are considered for initial pain control, regardless of the cause. In our case series, the most administered drug was carbamazepine at a dose of 800 mg/day. Additional second-line drugs typically include gabapentin (300-3600 mg/day) and pregabalin (150-600 mg/day). In our series, pregabalin, gabapentin, duloxetine, tramadol, and paracetamol were used. It is important to note that pharmacological treatment is not risk-free, and the most common adverse effects include dizziness, diplopia, ataxia, and hyponatremia [13-15].

**Table 3:** Trigeminal neuralgia treatment options. Pharmacological treatment is divided by first- and second-line indications and surgical options.

Treatment
Pharmacologic
First line: Carbamazepine, oxcarbazepine
Second line: Gabapentin, pregabalin, lamotrigine, phenytoin, baclofen, botulinum toxin type A
Surgical
Microvascular decompression: Janetta surgery
Rhizotomy percutaneous techniques: Balloon compression, radiofrequency, glycerol
Stereotactic radiosurgery
Deep brain stimulation
Peripheral technical: Local blocks (phenol, alcohol), neurectomy of branches

Surgical intervention for TN patients is necessary whenever adverse effects or symptoms are not abated with medical treatment. These options include MVD, PBC, percutaneous radiofrequency rhizotomy, percutaneous glycerol rhizotomy, and stereotactic radiosurgery. The most used techniques are MVD and PBC, which are strong, viable alternative options due to the low morbidity associated with these techniques. In general, all techniques have an initial response of more than 80% at five years [5].

MVD is a suboccipital craniotomy. It is the most invasive technique but has a superior response at a 10-year follow-up compared with the other percutaneous techniques. Significant risks associated with MVD occur at rates of less than 2% and include stroke, meningitis, and death, but in experienced hands, the complication rate is relatively low [2,5].

Radiofrequency rhizotomy provides a high initial success rate of 90%, though recurrence occurs in 25% of cases, with a long-term response rate of 57.7% at five years. PBC provides immediate pain relief in 80-90% of patients, with a long-term response rate of 69-80% at five years [1,2,16-18].

An interesting finding was that patients underwent surgery with 6.6 years of symptoms, probably due to low suspicion and low referral to specialists (neurologist or neurosurgeon). Furthermore, in the 10 years reviewed, we recorded a recurrence rate of 25%, which is compatible with the literature. These patients underwent a second surgery in an average of 2.7 years. 

PBC of the GG damages the thick myelinated fibers (which trigger pain by ephaptic-type contact with nociceptive fibers), usually leaving the thin and unmyelinated myelinated fibers unharmed [3,9,10]. Finally, the treatment effect may be caused by a voltage-gated sodium channel block, leading to the stabilization of hyperexcited neuronal membranes and inhibition of repetitive firing [6,10]. In 1954, Shelden and Pudenz concluded that patients with TN treated by decompression and compression of the peripheral branches in the oval and round foramen with a subtemporal approach had better results than decompression of the posterior root of the nerve (retro Gasserian section). These investigators concluded that compression of the trigeminal fibers during the operation was the prime factor that allowed for pain relief associated with decompression procedures [19]. Finally, in 1983, Mullan successfully described the actual technique of PBC of the ganglion through the foramen ovale. The advantages of the Mullan technique include a discomfort-free postoperative period, null mortality, and minimal morbidity [20]. Owing to its low complication incidence, it has advantages in elderly patients and can be repeated several times if symptoms continue to persist. Patients with atypical facial pain or postherpetic neuralgia are not viable candidates for this procedure.

During compression, pear-shaped balloon insufflation indicates adequate balloon pressure in the cavity. An hourglass image denotes balloon protrusion into the prepontine cistern. In such cases, the catheter is deflated, removed a few millimeters, and again insufflated. Exaggerated balloon insufflation compresses the upper inner wall of the cavum, with cranial nerve VI involvement. In our series, we observed a first-attempt, pear-shaped image in more than 90% of the patients.

Although percutaneous techniques are "less invasive," they also carry a risk of loss of sensitivity in the trigeminal territory, dysesthesia, loss of corneal reflex, painful anesthesia, meningitis, subdural hematoma, and a very low risk of mortality [2,21-24].

If a patient's pain is refractory to PBC performed using the proper technique, it is necessary for the practitioner to review the patient’s diagnosis. Patients with long-standing neuralgia and treatment-resistant trigeminal naturopathy can develop atypical facial pain.

## Conclusions

TN is a severely painful and disabling condition. Our 10-year experience with GG PBC highlights its efficacy after medical treatment failure. We observed high effectiveness and low morbidity, as evidenced by the absence of surgical complications and minimal symptomatic recurrence. While MVD remains the gold standard, our analysis suggests that PBC presents a compelling and easily implementable alternative for neurosurgical teams.

As a team, it seems interesting for future studies to protocolize the postoperative management of patients based on pain scales and local protocols in order to offer different surgical options depending on each patient. Even so, sufficient data have not yet been obtained and further studies are needed.
